# Immunohistochemical profiling of prognostic biomarkers in oral squamous cell carcinoma

**DOI:** 10.6026/973206300221394

**Published:** 2026-03-31

**Authors:** Amit Wasti, Shweta Chouhan, Vinod Sargaiyan, Rahul Raviraj Shetty, Lovna Titus, Vidhi Singhania, Miral Mehta

**Affiliations:** 1 Department of Oral Pathology and Microbiology, Government Dental College, Rajbandha Maidan, Raipur, Chhattisgarh, India; 2 Department of Oral Pathology and Microbiology, R.K.D.F. Dental College and Research Centre, Bhopal, Madhya Pradesh, India; 3 Department of Oral Pathology and Microbiology, Maharana Pratap College of Dentistry & Research Centre, Gwalior, Madhya Pradesh, India; 4 Department of Oral Medicine and Radiology, Bharati Vidyapeeth (Deemed to be University) Dental College and hospital, Navi Mumbai, India; 5 Department of Oral and Maxillofacial Surgery, Sri Sankara Dental College, Akathmuri, Thiruvananthapuram, Kerala, India; 6 Department of Oral Pathology & Microbiology, Sri Aurobindo College of Dentistry, Indore, Madhya Pradesh, India; 7 Department of Pediatric and Preventive Dentistry, Karnavati School of Dentistry, Karnavati University, Gandhinagar, Gujarat, India

**Keywords:** Oral squamous cell carcinoma (OSCC), immunohistochemistry, Ki-67, p53, epidermal growth factor receptor (EGFR), E-cadherin, cyclin D1, prognostic markers

## Abstract

Oral squamous cell carcinoma (OSCC) exhibits heterogeneous behavior, limiting the prognostic value of conventional histopathological grading alone. This study assessed Ki-67, p53, EGFR, E-cadherin and cyclin D1 expression in 120 formalin-fixed, paraffin-embedded OSCC specimens. High expression prevailed: Ki-67 (65.8%), mutant p53 (58.3%), EGFR (52.5%), reduced E-cadherin (60.0%), cyclin D1 (54.2%). Significant correlations emerged - Ki-67 with higher grade (p=0.001), nodal metastasis (p=0.008), advanced stage (p=0.012); E-cadherin loss with nodal spread (p=0.002). These markers serve as adjunctive prognostic biomarkers, improving OSCC risk stratification beyond routine histopathology.

## Background:

Oral squamous cell carcinoma is the most common malignancy that occurs in the oral cavity, with about 90-95 per cent of all oral malignancies and forms a considerable proportion of the world cancer burden [1]. Even with the current improvement in the diagnostic methodologies and multimodal approach in treating oral cancer, the five-year survival of this cancer is not optimal, as it depends on the stage, with the five-year survival of the cancer being between 50 and 65. This recurrent low prognosis puts emphasis on the urgent necessity of better knowledge of tumour biology and determination of strong prognostic factors [2]. Historical systems of histopathological grading, such as Broder classification and WHO grading, have been the classic standards of measuring tumour aggressiveness and their subsequent clinical behaviour. Nevertheless, these morphology-based methods have proven to be highly limited in terms of predicting patient outcome consistency since similar-looking tumours with similar histological features might have a very different clinical outcome [3]. Such a histological-clinical discrepancy indicates the heterogeneity of the diversity of the molecular details underlying the carcinogenesis of the oral cavity. The molecular pathogenesis of oral squamous cell carcinoma consists of dysregulations of various cell functions such as proliferation, apoptosis, cell cycle, cell adhesion and angiogenesis. Knowledge of such molecular changes opens possibilities of finding biomarkers that can be used to supplement traditional prognostic evaluation and make therapeutic decisions [4]. Immunohistochemistry provides a convenient, user-friendly approach that can be used to assess protein expression profiles in tissue samples to facilitate molecular characterisation in the general workflow of diagnostics. Ki-67 is a nuclear protein that is produced in active stages of the cell cycle and is used as an extensive proliferation marker. Ki-67 labelling index gives a quantitative evaluation of the proliferation percentage in tumour tissue and has been shown to have prognostic value in a variety of malignancies [5]. High levels of Ki-67 in oral cancer have been related to aggressive growth in tumours, although their relation to certain clinicopathological parameters remains to be explained. Tumour suppressor protein p53 is the key element in ensuring genomic stability by arresting the cell cycle, repairing DNA and inducing apoptosis. TP53 gene mutations are considered to be one of the most frequent genetic mutations observed in human cancers and the mutated p53 protein generally exhibits accumulation in the nucleus as revealed by immunohistochemistry [6].

The prognostic significance of p53 overexpression in oral cancer has been widely studied, but findings of reported associations with clinical outcomes are not consistent. Most of the head and neck squamous cell carcinomas over-express epidermal growth factor receptor (EGFR), which stimulates growth, survival, invasion and angiogenesis. EGFR has become a therapeutic target and cetuximab is an approved therapeutic target of head and neck cancer [7]. The description of EGFR expression patterns and clinical associations can be used in the determination of prognostic and therapeutic choices. E-cadherin is a calcium-dependent membrane glycoprotein that is involved in cell-cell adhesion of epithelial tissues and helps in tissue architecture maintenance. E-cadherin down regulation or loss of expression is a characteristic of epithelial-mesenchymal transition (EMT), which is linked to increased invasive ability and metastatic potential [8]. E-cadherin expression can thus be used to gain a clue on the invasive and metastatic potential of oral tumours. Cyclin D1 is a regulatory factor of the G1-to-S cell cycle transition of the cell and its overexpression has been associated with uncontrolled cellular proliferation. The 11q13 region can also be amplified to host the CCND1 gene and this amplification is common in head and neck malignancies and associated with the overexpression of the cyclin D1 protein [9]. The association between the expression of cyclin D1 and clinical variables in oral cancer is something that needs systematic research. Although there is an inquiry on the individual immunohistochemical markers in oral cancer, very few studies have analysed the marker panel and their associations with clinicopathological parameters, especially in Indian populations with different epidemiological and etiological features [10]. Therefore, it is of interest to assess the pattern of expression of Ki-67, p53, EGFR, E-cadherin and cyclin D1 in oral squamous cell carcinoma through the application of immunohistochemistry and to compare the expression levels with clinicopathological parameters such as tumour site, clinical stage, histological grade and lymph node status.

## Materials and Methods:

## Study design and setting:

This cross-sectional analytical study was conducted at the Department of Oral Pathology and Microbiology in collaboration with the Department of Oral and Maxillofacial Surgery between January 2021 and December 2023.

## Study population and sample selection:

The study included 120 patients with histopathologically confirmed oral squamous cell carcinoma who underwent surgical resection during the study period. Archived formalin-fixed paraffin-embedded (FFPE) tissue blocks from surgical specimens were retrieved for immunohistochemical analysis.

## Inclusion criteria:

[1] Histopathologically confirmed primary oral squamous cell carcinoma

[2] Primary tumour sites within the oral cavity (oral tongue, floor of mouth, buccal mucosa, gingiva, hard palate, retromolar trigone)

[3] Treatment-naive patients (no prior radiotherapy, chemotherapy, or surgical intervention)

[4] Adequate tissue availability in FFPE blocks

[5] Complete clinical and pathological records available

[6] Age ≥18 years

## Exclusion criteria:

[1] Recurrent oral squamous cell carcinoma

[2] Metastatic tumours to the oral cavity

[3] Verrucous carcinoma or other histological variants

[4] Prior neoadjuvant therapy

[5] Inadequate tissue for immunohistochemistry

[6] Oropharyngeal, hypopharyngeal, or laryngeal tumours

## Clinical data collection:

Clinical information was extracted from medical records, including patient demographics (age, gender), risk factor exposure (tobacco, alcohol, betel quid use), tumour characteristics (site, size, clinical stage according to AJCC 8th edition) and lymph node status. Follow- up data, including recurrence and survival status, were recorded when available.

## Histopathological evaluation:

All tissue specimens underwent comprehensive histopathological evaluation by two experienced oral pathologists.

Tumour grading was performed according to WHO criteria:

[1] Well-differentiated (Grade I): Closely resembling normal squamous epithelium with abundant keratinisation, minimal nuclear pleomorphism and rare mitoses

[2] Moderately differentiated (Grade II): Moderate degree of keratinisation, nuclear pleomorphism and mitotic activity

[3] Poorly differentiated (Grade III): Minimal keratinisation, marked nuclear pleomorphism and frequent mitoses

Histopathological parameters recorded included tumour depth of invasion, perineural invasion, lymphovascular invasion and status of surgical margins.

## Tissue processing for immunohistochemistry:

Representative tissue blocks containing adequate tumour tissue were selected for immunohistochemistry. Serial sections of 4 µ m thickness were cut and mounted on poly-L-lysine-coated slides. Sections were deparaffinized in xylene and rehydrated through graded alcohols.

## Antigen retrieval:

Heat-induced epitope retrieval was performed using the pressure cooker method. Sections were immersed in the appropriate retrieval buffer:

[1] Citrate buffer (pH 6.0) for Ki-67, p53, cyclin D1 and E-cadherin

[2] EDTA buffer (pH 9.0) for EGFR

Sections were heated at 120° for 20 minutes, followed by cooling to room temperature.

## Immunohistochemical staining:

Immunohistochemical staining was performed using the polymer-based detection system (EnVision FLEX, Dako, Agilent Technologies). The protocol included:

[1] Endogenous peroxidase blocking with 3% hydrogen peroxide for 10 minutes

[2] Primary antibody incubation for 60 minutes at room temperature

[3] Secondary polymer incubation for 30 minutes

[4] Chromogen development using diaminobenzidine (DAB) for 5-10 minutes

[5] Counterstaining with Mayer's hematoxylin

[6] Dehydration, clearing and mounting

## Primary antibodies:

The following primary antibodies were utilised:

[1] Ki-67 (Clone MIB-1, Dako): Ready-to-use

[2] p53 (Clone DO-7, Dako): Dilution 1:100

[3] EGFR (Clone E30, Dako): Ready-to-use

[4] E-cadherin (Clone NCH-38, Dako): Dilution 1:100

[5] Cyclin D1 (Clone EP12, Dako): Dilution 1:50

## Controls:

Positive controls included tonsillar tissue for Ki-67, a known p53-positive carcinoma for p53, placental tissue for EGFR, normal oral mucosa for E-cadherin and mantle cell lymphoma for cyclin D1. Negative controls were processed with the omission of the primary antibody.

## Immunohistochemical scoring:

Scoring was performed independently by two pathologists blinded to clinical data, with discordant cases resolved by consensus review.

## Ki-67 labelling index:

Percentage of positively stained tumour nuclei counted in five representative high-power fields (400x), with a minimum of 500 cells evaluated. Categorised as:

[1] Low: <10%

[2] Intermediate: 10-25%

[3] High: >25%

## p53 expression:

Nuclear staining intensity and percentage assessed:

[1] Negative/Wild-type pattern: <10% positive nuclei or variable weak staining

[2] Positive/Mutant pattern: ≥10% nuclei with strong diffuse staining

## EGFR expression:

Membranous staining scored according to intensity (0-3) and percentage of positive cells (0-100%). H-score calculated as: (1 x % weak) + (2 x % moderate) + (3 x % strong). Categorised as:

[1] Low expression: H-score <100

[2] Overexpression: H-score ≥100

## E-cadherin expression:

Membranous staining assessed for intensity and completeness:

[1] Preserved: Strong, complete membranous staining in >50% of tumour cells

[2] Reduced: Weak, incomplete, or absent staining in >50% of tumour cells

## Cyclin D1 expression:

Nuclear staining percentage:

[1] Negative/Low: <20% positive nuclei

[2] Overexpression: ≥20% positive nuclei

## Statistical analysis:

Statistical analysis was performed using SPSS version 26.0 (IBM Corporation, Armonk, NY). Continuous variables were expressed as mean ± standard deviation, while categorical variables were presented as frequencies and percentages. Associations between marker expression and clinicopathological parameters were analysed using the chi-square test or Fisher's exact test. Correlations between markers were assessed using Spearman's correlation coefficient. Inter-observer agreement for immunohistochemical scoring was evaluated using Cohen's kappa coefficient. Binary logistic regression identified independent predictors of marker expression. A two-tailed p-value <0.05 was considered statistically significant.

## Results:

The study cohort comprised 120 patients with a mean age of 54.8 ± 11.6 years (range: 28-78 years). Males predominate (72.5%), consistent with oral cancer epidemiology. Tobacco use was documented in 85.0% of patients, with chewing tobacco (58.3%) being more prevalent than smoking (42.5%). The buccal mucosa (35.8%) and oral tongue (30.0%) were the most common tumour sites. Demographic and clinical characteristics are presented in Table 1 (see PDF). High Ki-67 expression (labelling index >25%) was observed in 79 cases (65.8%), p53 over expression in 70 cases (58.3%), EGFR overexpression in 63 cases (52.5%), reduced E-cadherin expression in 72 cases (60.0%) and cyclin D1 overexpression in 65 cases (54.2%). Inter-observer agreement was substantial for all markers (κ=0.72-0.86). Significant associations were observed between marker expression and various clinicopathological features. Detailed correlation analysis is presented in Table 2 (see PDF). Inter-marker correlations were assessed using Spearman's correlation analysis. Significant positive correlations were observed between Ki-67 and p53 (r=0.42; p<0.001), Ki-67 and cyclin D1 (r=0.38; p<0.001) and p53 and cyclin D1 (r=0.35; p<0.001). Reduced E-cadherin expression showed a positive correlation with Ki-67 expression (r=0.34; p<0.001). EGFR expression demonstrated weak correlation with Ki-67 (r=0.28; p=0.002). Correlation analysis is summarised in Table 3 (see PDF).

Cases were stratified based on the number of adverse marker expressions (Ki-67 high, p53 positive, EGFR high, E-cadherin reduced, cyclin D1 high). Cases with ≥4 adverse markers demonstrated significantly higher rates of lymph node metastasis (82.4% vs. 47.7%; p<0.001), advanced clinical stage (85.3% vs. 51.2%; p<0.001) and poorly differentiated histology (44.1% vs. 14.0%; p<0.001) compared to cases with ≤3 adverse markers. Binary logistic regression analysis identified histological grade (OR=3.24; 95% CI: 1.86-5.64; p<0.001) and lymph node status (OR=2.18; 95% CI: 1.42-3.34; p<0.001) as independent predictors of high Ki-67 expression. Lymph node positivity (OR=2.86; 95% CI: 1.68-4.86; p<0.001) and tumour depth >10 mm (OR=2.12; 95% CI: 1.24-3.62; p=0.006) independently predicted reduced E-cadherin expression.

## Discussion:

This paper has undertaken the thorough determination of the expression of five immunohistochemical markers in oral squamous cell carcinoma and presented a significant correlation with clinicopathological parameters of aggressive tumour behaviour. The frequency of expression and correlation patterns observed will give a useful clue to the molecular nature of oral cancer in this group of patients. A high level of 65.8% of Ki-67 expression in our cohort is comparable to the results of the previous studies on the proliferation markers studied in oral cancer. Ki-67 labelling indices have been reported in the population studies to be 40-80% in OSCC, with variations in these levels being due to methodology differences and definitions of score cutoff [11]. The close relationship between large Ki-67 levels and low levels of histological differentiation indicates the essence of the relationship between proliferative action and loss of the ability to differentiate in malignant cells. The relationship between Ki-67 and lymph node metastasis, as observed in our study, confirms the idea that increased proliferative ability is one of the factors that form metastatic potential. Growing tumours can have an increased supply of cells that can enter the lymphatic system and create nodal metastases. Meta-analyses done in the past have validated Ki-67 as an important prognostic factor in squamous cell carcinoma of the head and neck [12]. Our study results of p53 overexpression of 58.3 per cent are within the range of generally reported p53 overexpression in oral cancer. Detection of p53 by immunohistochemistry typically indicates the presence of mutant protein with a half-life that is extended half-life, but under specific conditions of the wild type of stabilization, false-positive results can be obtained [13]. The correlation between a positive p53 and increased histological grade is in line with the importance of the p53 dysfunction in allowing genomic instability and progressive dedifferentiation. The correlation between the p53 expression and the lymph node status that we have revealed, in our analysis, can give support to the functional role of p53 changes in the determination of metastatic behaviour. Due to loss of p53 tumour suppressor activity, cells with cumulative genetic damage, which would otherwise have been subjected to apoptosis, would be able to survive, which may in turn select clones with superior invasive and metastatic traits [14]. The 52.5% overexpression of EGFR in our cases is comparable to the literature, which indicates that EGFR is positive in 40-90 per cent of squamous cell carcinomas of the head and neck. The correlation between the expression of EGFR and tumour size should indicate that EGFR signalling has a growth-stimulating effect because it activates the downstream pathways like RAS-MAPK and PI3K-AKT that mediate cell growth and survival [15]. EGFR expression has clinical implications due to the wider theoretical applications of EGFR-targeted therapeutics such as cetuximab in the treatment of head and neck cancer. The decreased expression of E-cadherin in 60.0% of the cases, along with the high correlation with lymph node metastasis, is a rather important fact. The down-regulation of e-cadherin breaks intercellular adhesion, which promotes the detachment of tumour cells, invasion of the stroma and metastatic spread. Such a molecular change is a characteristic of epithelial-mesenchymal transition, a program that is becoming central to cancer invasion and metastasis [16]. The loss of E-cadherin and poorly differentiated histology is associated with the role of E- cadherin in ensuring epithelial differentiation phenotype. The cells in epithelial-mesenchymal transition change their epithelial features, acquiring mesenchymal features and histologically changing to a loss of differentiated morphology [17]. The potential prognostic value of E-cadherin status may be highlighted by the independent predictive value of E-cadherin status on lymph node metastasis in multivariate analysis. Overexpression of Cyclin D1 in 54.2 per cent of cases is supported by the common amplification of the 11q13 chromosomal region of oral cancer. The direct mechanistic relationship is indicated by the correlation between the expression of cyclin D1 and the Ki-67 labelling index because the cyclin D1 causes G1-to-S phase transition, which augments cellular proliferation [18]. Its connection with lymph node positivity and high stage is another indicator that demonstrates the poor prognostic outcomes of cyclin D1 overexpression. The high inter-marker correlations that were identified in this study indicate the inter-relationship of the cellular regulatory pathways. It is mechanistically predictable that Ki-67 and cyclin D1 have a positive correlation, since both of them are associated with cell cycle progression. This correlation of p53 and cyclin D1 can indicate the release of the p53-regulated cell cycle in the presence of the mutated p53 and thus further enhance the proliferative impact of the cyclin D1 overexpression [19]. The joint marker system with poorer clinicopathological characteristics in cases that have many cases of poor adverse marker expressions indicates that composite biomarker panels may have some potential uses. Single markers are only partially prognostic and an integrated assessment can help to better describe tumour biology. Other multi-marker strategies have demonstrated improved prognostic capabilities in multiple malignancies [20]. Clinical utility of immunohistochemical markers in the management of oral cancers is not only limited to prognostication but also to a treatment plan. The presence of high-risk molecular features has the potential to identify those patients who respond to the intensified adjuvant therapy, whilst low-risk profiles can help de-escalate treatment in the right situations. Also, EGFR can be used to guide the selection of targeted therapy [21]. Tobacco-related oral cancer is predominant in our population and this could have an effect on the marker expression patterns than in cohorts with other etiological profiles. The tobacco carcinogens cause certain mutational signatures that can have varying impacts on the molecular pathways investigated. Also, the anatomical distribution that is mostly contributed by the buccal mucosa indicates the regional habits and can be considered to affect the generalizability of the findings in populations with varied distributions of the sites [22]. Issues such as tissue fixation, selection of antibodies and scoring systems all affect the outcomes of immunohistochemical staining and make cross-study comparisons difficult. High inter-observer agreement obtained in our study indicates the reliability of the methods used, but standardisation between laboratories is problematic. Digital image analysis can provide a better reproducibility in future research [23]. These are some of the limitations that should be mentioned. The cross-sectional study design does not allow a direct survival analysis; a prospective follow-up would enhance prognostic inferences. There is a risk that the cohort of single institutions may not be a good reflection of oral cancer heterogeneity. HPV status, which is becoming an important factor in head and neck cancer, was not evaluated. Moreover, the chosen marker panel, as comprehensive as it is, does not cover the possibly relevant biomarkers exhaustively.

## Conclusion:

We show Ki-67, p53, EGFR, E-cadherin and cyclin D1 are strongly associated with aggressive clinicopathological features in oral squamous cell carcinoma, particularly high Ki-67 and low E-cadherin with nodal metastasis, advanced stage and poor differentiation. Observed inter- marker relationships suggest interconnected molecular pathways regulating proliferation, cell cycle control and epithelial-mesenchymal transition, with combined adverse marker profiles identifying cases with markedly worse phenotypes. Thus, we show adding immunohistochemical marker evaluation to routine histopathology to refine prognostic stratification, guide treatment intensity and inform future prospective studies validating their role in clinical decision-making.
